# Type 1 and type 2 cytokine-mediated immune orchestration in the tumour microenvironment and their therapeutic potential

**DOI:** 10.37349/etat.2023.00146

**Published:** 2023-06-30

**Authors:** Eric Jou

**Affiliations:** University of Kansas Medical Center, USA; ^1^Queens’ College, University of Cambridge, CB3 9ET Cambridge, UK; ^2^MRC Laboratory of Molecular Biology, CB2 0QH Cambridge, UK

**Keywords:** Tumour microenvironment, cancer therapy, targeted therapy, preclinical models, immunotherapy, cytokines, oncology trials

## Abstract

Cancer remains the second leading cause of death worldwide despite modern breakthroughs in medicine, and novel treatments are urgently needed. The revolutionary success of immune checkpoint inhibitors in the past decade serves as proof of concept that the immune system can be effectively harnessed to treat cancer. Cytokines are small signalling proteins with critical roles in orchestrating the immune response and have become an attractive target for immunotherapy. Type 1 immune cytokines, including interferon γ (IFNγ), interleukin-12 (IL-12), and tumour necrosis factor α (TNFα), have been shown to have largely tumour suppressive roles in part through orchestrating anti-tumour immune responses mediated by natural killer (NK) cells, CD8^+^ T cells and T helper 1 (Th1) cells. Conversely, type 2 immunity involving group 2 innate lymphoid cells (ILC2s) and Th2 cells are involved in tissue regeneration and wound repair and are traditionally thought to have pro-tumoural effects. However, it is found that the classical type 2 immune cytokines IL-4, IL-5, IL-9, and IL-13 may have conflicting roles in cancer. Similarly, type 2 immunity-related cytokines IL-25 and IL-33 with recently characterised roles in cancer may either promote or suppress tumorigenesis in a context-dependent manner. Furthermore, type 1 cytokines IFNγ and TNFα have also been found to have pro-tumoural effects under certain circumstances, further complicating the overall picture. Therefore, the dichotomy of type 1 and type 2 cytokines inhibiting and promoting tumours respectively is not concrete, and attempts of utilising these for cancer immunotherapy must take into account all available evidence. This review provides an overview summarising the current understanding of type 1 and type 2 cytokines in tumour immunity and discusses the prospects of harnessing these for immunotherapy in light of previous and ongoing clinical trials.

## Introduction

Despite modern medical breakthroughs, cancer is now the second leading cause of death worldwide [1], and global cancer incidence is projected to increase by a further 50% in the next 20 years [2]. The urgent development of novel cancer therapies is a key aspect of contemporary medicine and is more vital than ever. Whilst cancer immunotherapy, in particular immune checkpoint inhibitors which act by revitalising T cells, have completely revolutionised cancer treatment resulting in immunotherapy being erected as the fourth pillar of cancer treatment alongside traditional surgery, chemotherapy, and radiotherapy, not all cancer types or patients respond [3–5]. Identifying novel cancer immunotherapies to overcome therapeutic resistance is challenging yet crucial, and currently, two-thirds of ongoing clinical trials in oncology involve immunotherapy [5].

Cytokines are small secreted proteins (6–70 kDa) produced by a broad range of immune and non-immune cell types allowing intercellular communication with key roles in orchestrating the immune response [6]. During protective immunity against pathogens, the specific types and combinations of cytokines released are tightly coordinated in order to elicit the optimal immune response for pathogen clearance [7, 8]. Similarly, the immune system is able to actuate cancer immunosurveillance and tumour rejection through an array of anti-tumour cytokines and effector immune cells [9]. However, established malignancies are able to create a tumour microenvironment that supports neoplastic growth through the release of immunomodulatory cytokines [10], and indeed, immunoevasion is a key hallmark of cancer as described by Hanahan and Weinberg [11].

**Figure 1:**
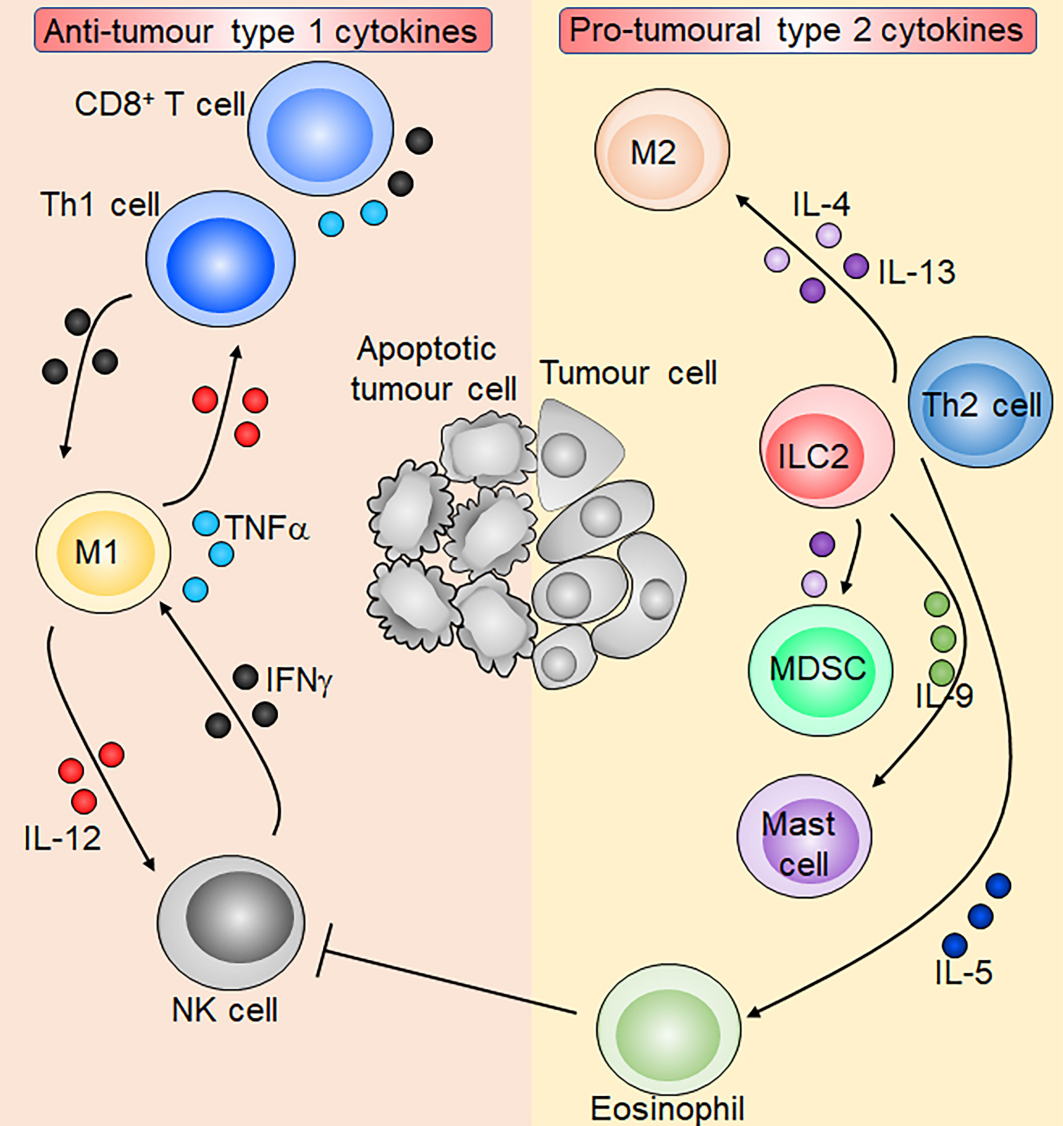
Overview of type 1 and type 2 cytokines in tumour immunity. M1 macrophages produce IL-12 which activates NK cells and CD8^+^ T cells and induces Th1 cell polarisation. Activated NK cells, CD8^+^ T cells, and Th1 cells in turn produce IFNγ which reciprocally activates M1 macrophages. T cells and M1 macrophages are also capable of producing TNFα. ILC2s and Th2 cells produce the type 2 cytokines IL-4, IL-5, IL-9, and IL-13. IL-4 and IL-13 induce M2 macrophages and immunosuppressive myeloid-derived suppressor cells (MDSCs), which inhibit anti-tumoural T cells. IL-5 and IL-9 promote eosinophils and mast cells respectively, and eosinophils may inhibit NK cell function. Overall, type 1 cytokines orchestrate an anti-tumour immune response leading to tumour cell apoptosis, while type 2 cytokines promote tumorigenesis. Sharp arrows indicate activation or stimulation while blunted arrows indicate inhibition. Figure was created in part using cartoon templates by Servier Medical Art (https://smart.servier.com/), licensed under a Creative Commons Attribution 3.0 Unported License

The immune system can be broadly classified into opposing type 1 and type 2 immunity each involving a unique array of cytokines with distinct functions [7, 12]. The type 1 immune cytokines interferon γ (IFNγ), interleukin-12 (IL-12), and tumour necrosis factor α (TNFα) orchestrate the protective type 1 immunity consisting of natural killer (NK) cells, CD8^+^ cytotoxic T cells, and CD4^+^ T helper 1 (Th1) cells, against intracellular pathogens such as viruses or intracellular bacteria [7, 13, 14]. On the other hand, the type 2 immune response has important roles in anti-helminth immunity, tissue regeneration, and wound repair, and is orchestrated by group 2 innate lymphoid cells (ILC2s) and Th2 cells with effector functions driven by the cytokines IL-4, IL-5, IL-9, and IL-13 [8, 15, 16]. Traditionally, type 1 immunity has been ascribed a predominantly anti-tumoural role showcased by many preclinical and clinical studies, while type 2 immunity harboured by the tumour microenvironment is associated with wound healing and repair thereby creating a pro-tumorigenic niche (Figure 1) [17–25]. Nevertheless, recent studies indicate that the dichotomy between type 1 and type 2 cytokines in tumour immunity is not concrete as previously envisaged [26–29]. Under certain contexts, type 1 immunity may promote cancer development through chronic inflammation, while type 2 cytokines may elicit anti-tumour immunity through blood vessel remodelling and macrophage recruitment [26–29]. Therefore, any attempts to modify the tumour microenvironment through cytokine-based immunotherapy must be carefully tailored to the specific context in order to achieve the desired anti-tumoural effect.

This review provides an overview of the current understanding of the classical type 1 and type 2 cytokines in the tumour microenvironment, with particular focus on their role in tumour immunity. The type 2 immunity-related cytokines IL-25 and IL-33 with recently discovered roles in cancer are also explored in light of their potential as novel immunotherapeutic targets [30, 31]. Existing cytokine-based cancer immunotherapies and the broader prospects of harnessing cytokines for future cancer treatments are also discussed.

## Type 1 cytokines in tumour immunity

In general, cytokines involved in type 1 immunity including IFNγ, IL-12, and TNFα are shown to orchestrate anti-tumour immune responses against a broad range of cancer types in numerous preclinical and clinical studies [32]. IFNγ is the main effector cytokine of type 1 immune response and is established as the prototypical anti-tumoural cytokine with critical roles in anti-cancer immunity and tumour rejection [33]. IL-12 mainly exerts function through inducing IFNγ expression [34, 35], while TNFα has conflicting roles and may either promote or suppress tumorigenesis [36].

### IFNγ

**Figure 2:**
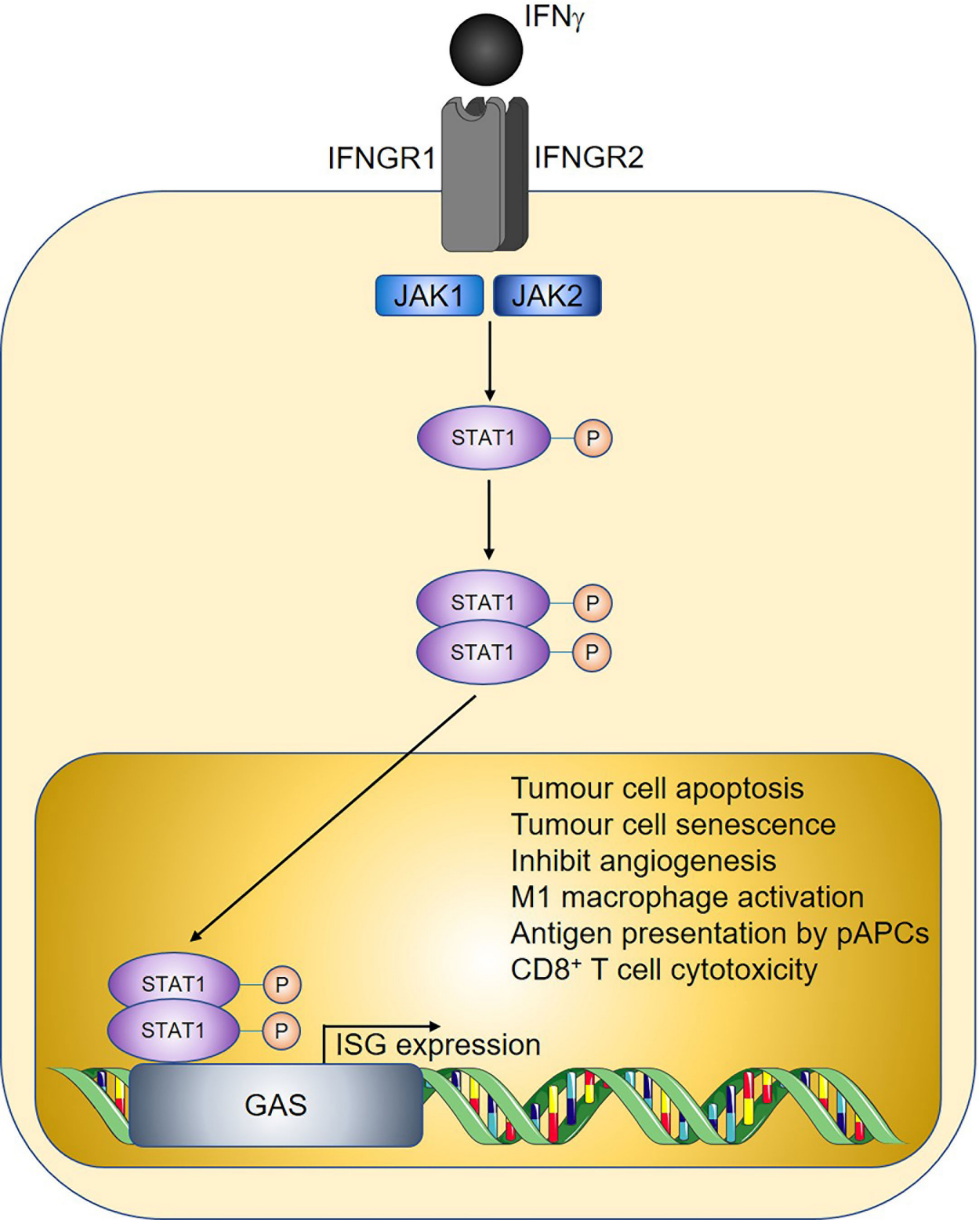
Overview of IFNγ signalling and anti-tumoural properties. Sharp arrows indicate activation; circled P symbols indicate phosphorylation. Figure was created in part using cartoon templates by Servier Medical Art (https://smart.servier.com/), licensed under a Creative Commons Attribution 3.0 Unported License

IFNs are cytokines with important roles in antiviral immunity and consist of three major families, designated as type I, II, and III [37]. Whilst type I and type III IFNs predominantly protect the host from pathogens, IFNγ being the sole member of type II IFNs, has additional well-documented functions in cancer immunosurveillance [38–41]. In the tumour microenvironment, sources of IFNγ include tumour-infiltrating NK cells, ILC1s, γδ T cells, CD8^+^ T cells, and Th1 cells [42–44]. IFNγ signaling is mediated by the IFNγ receptor (IFNGR), leading to downstream activation of Janus kinase 1 (JAK1) and JAK2, respectively, and subsequent phosphorylation of the transcription factor signal transducer and activator of transcription 1 (STAT1) [45]. Nuclear translocation of the phosphorylated STAT1 homodimer then ensues, allowing subsequent binding to the corresponding gamma-activated sequence (GAS) DNA element and induction of IFN-stimulated gene (ISG) expression (Figure 2).

IFNγ signaling in cancer cells directly results in apoptosis through the induction of an array of pro-apoptotic genes including absent in melanoma 2 (*AIM2*) gene, IFN-induced transmembrane protein 2 (*IFITM2*) gene, inositol hexakisphosphate kinase 2 (*IP6K2*) gene, *ISG12a*, *ISG54*, phorbol-12-myristate-13-acetate-induced protein 1 (*NOXA*) gene, and neuronal precursor cell-expressed developmentally down-regulated protein 8 (NEDD8) ultimate buster 1 (*NUB1*), which ultimately results in a reduction in antiapoptotic B-cell lymphoma-2 (BCL-2) proteins and downstream activation of caspase 3 leading to cell death [46, 47], and IFNγ has also been shown to promote cell death via induction of nitric oxide (NO) or reactive oxygen species (ROS)-mediated pathways [48]. Conversely, IFNγ binding to receptors on anti-tumour type 1 immune cells typically results in activation and induction of effector function [49]. Accordingly, in mouse models of colorectal cancer (CRC), heterozygous genetic deletion of IFNγ (*Ifng*^+/-^), homozygous deletion of the corresponding receptor (*Ifngr1*^-/-^), or antibody-mediated neutralisation of IFNγ, increased adenocarcinoma development [24, 41, 50]. In humans, an *IFNG*-associated type 1 immune signature is associated with improved prognosis in human CRC patients [51], and similarly predicts prolonged patient survival across multiple other cancer types such as breast, liver, and ovarian cancers [52–54]. Mechanistically, IFNγ induces the expression of cytokines IL-1β, IL-6, IL-12, IL-18, IL-23, TNFα, and proinflammatory factors such as ROS and NO by M1 macrophages, thereby contributing to M1 macrophage-mediated anti-tumour immunity [55, 56]. Both IFNγ and TNFα promote M1 macrophage polarisation [57, 58], and induction of M1 macrophages is associated with reduced tumour burden in mouse CRC [59]. Similarly in humans, increased tumour M1 macrophage infiltration is associated with improved survival in multiple cancer types including ovarian [60], lung [61], and hepatocellular carcinoma [62].

Furthermore, IFNγ stimulates major histocompatibility complex class II (MHC-II) expression on professional antigen-presenting cells (pAPCs) such as dendritic cells (DCs) and macrophages through the transcriptional coactivator MHC class II transactivator (CIITA) [63], and together with M1 macrophage-derived IL-12, promotes T cell activation and subsequent polarisation towards a Th1 phenotype [64, 65]. As mentioned, Th1 cells are potent producers of IFNγ thus providing positive feedback, and both Th1 cells and IFNγ play critical roles in CD8^+^ cytotoxic T cell activation, the latter directly while the former indirectly through licensing XC chemokine receptor 1^+^ (XCR1^+^) DCs [43, 66]. XCR1^+^ DCs are specialised in cross-presentation of antigens, which is critical in efficient CD8^+^ T cell activation [67]. Whilst CD8^+^ T cells, like Th1 cells, are potent producers of IFNγ, they additionally exert direct cytotoxicity on tumour cells through Fas ligand (FasL) or perforin and granzyme which can be further enhanced in response to IFNγ signalling [68]. Cytotoxic CD8^+^ T cells have well-established roles in eliminating cancer cells *in vivo* [69], and are the most potent anti-tumour immune cell type known against human cancer [70].

Various other mechanisms of IFNγ-mediated anti-tumour immunity have been proposed (Figure 2), for example by decreasing cancer stem cells in the 4T1 mouse model of breast cancer [43, 71]. IFNγ induces cancer cell senescence and arrest via activating p16^Ink4a^/p19^Arf^ and p21^Cip1^ thereby inhibiting tumorigenesis in models of pancreatic islet cancer and Burkitt lymphoma [72]. Furthermore, in a heterotypic xenograft mouse model of human gallbladder cancer, treatment with IFNγ reduced tumorigenesis by inhibiting angiogenesis. This is via suppression of M2 macrophage vascular endothelial growth factor (VEGF) production and highlights the therapeutic potential of IFNγ [73]. Overall, IFNγ is capable of inducing anti-tumour immunity via direct action on tumour cells or indirectly through stimulating type 1 immunity.

### IL-12

IL-12 is part of the IL-12 family of cytokines which includes IL-23, IL-27, IL-35, and IL-39, each with very distinct functions [74, 75]. For instance, whilst IL-12 is crucial in Th1 polarisation through the induction of STAT4 and T-box expressed in T cells (Tbet) thereby eliciting anti-tumour immunity [65], IL-23 participates in Th17 immune responses [76] while IL-35 is a recently characterised cytokine with immunosuppressive and pro-tumorigenic properties [77]. IL-12 is produced by the pAPCs macrophages and DCs, and signalling is mediated through binding to the IL-12 receptor (IL-12R), a heterodimer consisting of IL-12Rβ1 and IL-12Rβ2, on target cells [78]. Mechanistically, IL-12 exerts potent anti-tumoural functions by coordinating the aforementioned anti-tumour type 1 response and suppresses alternative T cell fates by inhibiting Th2, Th17, and regulatory T (Treg) cell differentiation [34, 35]. IL-12-induced Th1 cells in turn activate NK cells and CD8^+^ T cells, all of which are potent IFNγ producers [79]. IFNγ can further enhance IL-12 production by pAPCs thereby creating a positive feedback loop [80]. Mice genetically deficient in functional IL-12 are more susceptible to skin cancer and sarcoma development [81, 82], while treatment with recombinant IL-12 delayed tumorigenesis, reduced peritoneal seeding, and prolonged survival in mice with metastatic ovarian cancer [83]. IL-12 can also induce IFNγ-independent CRC tumour rejection via activation of CD8^+^ T cells, a process that is dependent on CD4^+^ T cells and granulocyte-macrophage colony-stimulating factor (GM-CSF) [84]. Similarly, in humans, polymorphisms in the IL-12 genes *IL-12B* (encoding the p40 subunit of IL-12, A > C, rs3212227), are associated with increased risk of breast cancer when harbouring the A allele [odds ratio (OR) = 1.68, 95% confidence interval (CI) 1.09–2.59] [85], while the *IL-12A* rs568408 GA/AA variant is associated with increased risk of cervical cancer (OR = 1.43, 95% CI 1.06–1.93) [86].

### TNFα

On the other hand, unlike IL-12 and IFNγ with predominant anti-tumoural properties, the role of TNFα in tumorigenesis is context-dependent. Whilst initially discovered as an anti-tumoural cytokine capable of inducing necrosis of sarcomas in mice [87], more recent studies have uncovered pro-tumoural aspects of TNFα [36]. TNFα is produced by macrophages (similar to IL-12), and additionally by NK cells and activated T cells. TNFα elicits antigen-independent killing of tumour cells by CD8^+^ T cells and NK cells [88], and can directly induce target cell death through signalling [89, 90]. Conversely, genetic deficiency of TNFα enhanced tumour burden in a mouse model of skin cancer [91], and in humans elevated TNFα expression is associated with breast cancer recurrence [92] and poor prognosis in ovarian cancer patients [93]. TNFα may promote tumours through various proposed mechanisms. TNFα may promote breast cancer cell stemness through the nuclear factor kappa B (NF-κB) pathway [94, 95], or through inducing dedifferentiation of melanoma cells resulting in downregulation of tumour antigens thereby promoting immunoevasion [96]. Furthermore, TNFα may directly promote Treg activation through the TNF receptor type 2 (TNFR2) thereby suppressing anti-tumour immunity [97].

## Type 1 cytokines in immunotherapy

Type 1 immune cytokines are detected in a broad range of cancer types with well-established prognostic roles and are therefore attractive therapeutic targets for cancer treatment [36, 98–100]. Due to its potent anti-tumoural functions, single agent IFNγ therapy was tested in some clinical trials in the 1990s for cancer treatment however showed largely inconsistent results [101]. Given that the tumour microenvironment is highly immunosuppressive with complex interactions between tumour cells, stroma, and infiltrating immune cells, single-agent therapies are unlikely to be adequate in overcoming tumour immune evasion [102]. Indeed, most modern clinical trials involving IFNγ largely focus on combinational therapies to overcome therapeutic resistance. In phase III clinical trial of 148 patients with the International Federation of Gynecology and Obstetrics (FIGO) stage Ic–IIIc ovarian cancer, the addition of subcutaneous IFNγ treatment to the combined cisplatin and cyclophosphamide regime improved progression-free survival (PFS) to 51% in the treatment arm compared to 38% in the control group [103]. This trial serves as a proof of concept demonstrating that IFNγ can be utilised in the treatment of cancer. Furthermore, in a phase II trial of patients with ovarian, fallopian tube, and primary peritoneal cancer, the addition of IFNγ and GM-CSF to standard carboplatin treatment led to patient-reported improvements in quality of life [104].

However, a phase II trial (NCT00786643) adding IFNγ to the 5-fluorouracil, leucovorin, and bevacizumab regimen for metastatic CRC showed no additional benefits. Importantly, recent studies have uncovered potential pro-tumoural functions of IFNγ, with proposed mechanisms including induction of immunosuppressive programmed death-ligand 1 (PD-L1) and indoleamine 2,3-dioxygenase (IDO) expression, along with induction of CD8^+^ T cell apoptosis [105]. Further studies are required to understand the specific conditions that render IFNγ pro-tumoural. Critically, immunotherapeutic treatment with IFNγ must be carefully designed to maximise efficacy and to prevent iatrogenic harm through tumour induction, especially as all upcoming and ongoing clinical trials continue to utilise IFNγ as a potential treatment agent for cancer (Table 1). Furthermore, IFNγ treatment may be associated with toxicities for example through overactivation of macrophages. In a pilot trial where two patients with synovial sarcoma were treated with an addition of IFNγ and IL-2 to the combined cyclophosphamide and adoptive T cell transfer therapy, although one patient showed significant tumour regression, the other suffered fatal histiocytic myocarditis [106]. Studies on human melanoma biopsies found an IFNγ signature to be associated with a high response to checkpoint inhibitor therapy in patients, and *in vitro* exposure of 58 distinct human cell lines to IFNγ induced a similar signature [107]. This indicates that combinational treatment with IFNγ may potentiate immune checkpoint inhibitor efficacy, and this exciting prospect will be tested in upcoming trials (Table 1).

**Table 1:** A comprehensive list of ongoing oncology clinical trials involving IFNγ and IL-12, either alone or in combination with other cytokine-based therapies

**Cytokine**	**Clinical trial ID**	**Phase**	**Number of patients**	**Cancer type**	**Treatment**	**Status**	**Estimated study completion date**
IFNγ	NCT03112590	I/II	51	HER2^+^ breast cancer	IFNγ plus paclitaxel, trastuzumab and pertuzumab	Active	June 2023
IFNγ	NCT03063632	II	28	Mycosis fungoides, Sezary syndrome, and advanced synovial sarcoma	IFNγ plus pertuzumab	Active	April 2023
IFNγ	NCT05268172	I	40	Malignant pleural effusion tumours	IFNγ plus T cells	Recruiting	December 2023
IFNγ	NCT03747484	I/II	16	Metastatic or unresectable Merkel cell cancer	IFNγ plus T cells and avelumab or pembrolizumab	Recruiting	December 2025
IL-12	NCT02555397	I	15	Prostate cancer	Adenovirus-mediated cytotoxic and *IL-12* gene therapy	Active	February 2023
IL-12	NCT04491955	II	23	Small bowel cancer and CRC	NHS-IL-12 plus CV301 vaccine, M7824 (anti-PD-L1/TGFβ Trap fusion protein) and N-803 (IL-15 superagonist) combination immunotherapy	Active	July 2024
IL-12	NCT03439085	II	77	HPV associated malignancies	DNA plasmid-encoding IL-12/HPV DNA plasmids vaccine (MEDI0457) plus durvalumab	Active	December 2022
IL-12	NCT04015700	I	9	Glioblastoma	Plasmid encoded IL-12 with personalised neoantigen DNA vaccine	Active	April 2023
IL-12	NCT04911166	I	16	Metastatic non-small cell lung cancer	Adenovirus-mediated *IL-12* gene therapy plus atezolimumab	Recruiting	June 2024
IL-12	NCT05162118	I/II	51	Advanced pancreatic cancer	VG161 (IL-12/IL-15/PD-L1 blocking peptide) oncolytic HSV1 injection plus nivolumab	Recruiting	December 2025
IL-12	NCT04287868	I/II	51	Advanced HPV-associated malignancies	NHS-IL-12 plus PDS0101 and M7824 (anti-PD-L1/TGFβ Trap fusion protein)	Recruiting	January 2024
IL-12	NCT05392699	I	60	Advanced solid tumours	Human *IL-12* mRNA	Recruiting	January 2027
IL-12	NCT04806464	I	44	Primary liver cancer	IL-12/IL-15/PD-L1 blocking peptide oncolytic HSV1 injection	Recruiting	December 2022
IL-12	NCT04708470	I/II	90	HPV-associated malignancies, small bowel cancer, and CRC	NHS-IL-12 plus bintrafusp alfa and entinostat	Recruiting	December 2024
IL-12	NCT04471987	I	94	Advanced or metastatic solid tumours	IL-12-L19L19	Recruiting	December 2023
IL-12	NCT04388033	I/II	10	Glioblastoma	IL-12 plus DC tumour vaccine and temozolomide	Recruiting	December 2023
IL-12	NCT02483312	I	9	Acute myeloid leukemia	IL-12	Recruiting	February 2022
IL-12	NCT04758897	I	18	Advanced malignant solid tumours	VG161 (IL-12/IL-15/PD-L1 blocking peptide) oncolytic HSV1 injection	Recruiting	December 2022
IL-12	NCT05477849	I	30	Advanced malignant solid tumours	IL-12/IL-15 dual-regulated oncolytic HSV1 injection	Recruiting	December 2024
IL-12	NCT04303117	I/II	64	Kaposi sarcoma	NHS-IL-12 with or without M7824 (anti-PD-L1/TGFβ Trap fusion protein)	Recruiting	December 2025
IL-12	NCT05717712	I	18	Glioma	Adenovirus-mediated-non-secretory-IL-12	Recruiting	January 2028

HER2: human epidermal growth factor receptor 2; TGFβ: transforming growth factor β; HPV: human papillomavirus; HSV1: herpes simplex virus 1; mRNA: messenger RNA

IL-12 has been considered a strong candidate for cytokine-based immunotherapy due to its potent properties in promoting anti-tumour type 1 immunity and IFNγ responses [108]. However, direct systemic administration of IL-12 is associated with significant toxicities. In a phase 2 study of 17 patients with advanced renal cell carcinoma, systemic administration of IL-12 led to severe toxicities resulting in 12 patients being hospitalised and two deaths likely due to overwhelming IFNγ release [109]. In phase I/II trial of 33 patients with metastatic melanoma, autologous transfer of tumour infiltration lymphocytes transduced with the gene encoding IL-12 showed enhanced anti-tumour efficacy, but was associated with significant toxicities including high fevers, liver dysfunction, and life-threatening haemodynamic instability [110]. Potential alternative methods to deliver IL-12 are being developed through preclinical models in an attempt to improve safety, for example through nanoparticles [111]. In an animal model of pancreatic cancer, oncolytic virus-mediated delivery of IL-12 which lacks the signalling peptide enhanced survival while inducing minimal toxicities [112]. Altogether, overcoming toxicities in upcoming clinical trials remains the major challenge of implementing IL-12 for cancer treatment (Table 1).

Methods to further improve the efficacy of IL-12-based therapies are being tested. As both IFNγ and IL-12 activate T cells, adoptive transfer of T cells with co-administration of these cytokines may enhance anti-tumour immunity and prevent T cell exhaustion as seen in mouse models of melanoma [113, 114]. The T cell activating properties of IL-12 may further allow synergy with checkpoint inhibitors. NHS-IL-12, a recombinant fusion protein consisting of IL-12 fused to the human monoclonal immunoglobulin G1 (IgG1) antibody NHS76, has been shown to have improved tumour targeting abilities and longer plasma half-life compared to IL-12, resulting in further enhanced activation of pAPCs and reduction in tumour growth in a mouse model of bladder cancer [115]. These are all being investigated in ongoing clinical trials with exciting prospects (Table 1).

Finally, whilst TNFα has well-established roles in promoting or inhibiting tumorigenesis, the majority of existing trials to date focussed on enhancing TNFα signalling in cancer treatment. This is based on the rationale that high levels of TNFα promote tumour rejection, while chronic low levels of sustained TNFα expression, as seen in the tumour microenvironment, instead promotes tumorigenesis [36]. Indeed, preclinical studies support the notion of exogenous TNFα inhibiting tumorigenesis. In a mouse xenograft model of human breast cancer, TNFα enhanced the cytotoxicity exerted by combined chemotherapy with docetaxel, 5-fluorouracil, and cisplatin [116]. However, the majority of clinical trials involving systemic TNFα treatment were limited by sepsis-like symptoms and showed no efficacy in tumour rejection at the maximally tolerated dose in phase I and phase II trials [117–119]. A potential alternative to boost TNFα signalling and improve the safety profile is through the removal of the inhibitory plasma soluble TNFR rather than direct administration of TNFα, and ongoing clinical trials that explore this are listed in Table 2. Similar to IL-12-based therapies, reducing therapeutic toxicities is vital for the future success of utilising TNFα in cancer treatment.

**Table 2:** All ongoing oncology clinical trials involving the removal of soluble TNFR

**Cytokine**	**Clinical trial ID**	**Phase**	**Number of patients**	**Cancer type**	**Treatment**	**Status**	**Estimated study completion date**
TNFα	NCT04004910	I/II	170	Advanced breast cancer	Three-way comparison between plasma soluble TNFR pulldown with or without chemotherapy, and chemotherapy alone	Recruiting	July 2023
TNFα	NCT04142931	I	30	Stage IV non-small cell lung cancer, stage IV melanoma, triple-negative breast cancer, or stage IV renal cell carcinoma	Reduction of soluble TNFR, with or without nivolumab	Recruiting	December 2022
TNFα	NCT04690686	II	24	Non-small cell lung cancer	Reduction of soluble TNFR alone, or with atezolizumab or paclitaxel	Recruiting	December 2022

## Type 2 cytokines in tumour immunity

The key cytokines that partake in type 2 immunity include IL-4, IL-5, IL-9, and IL-13 with critical roles in protective anti-helminth immunity, wound healing, and tissue regeneration [8, 15, 16]. ILC2s are recently discovered tissue-resident cells found mainly at epithelial and mucosal barriers that rapidly respond to acute immune perturbations [120] and represent the dominant early innate source of these cytokines [13, 121, 122]. Within the innate immune system, mast cells and eosinophils may also contribute to IL-4 and IL-13 production [123–125]. In adaptive immunity, Th2 cells are the major source of IL-4, IL-5, and IL-13 while the closely related Th9 cells produce IL-9, thereby amplifying the type 2 immune response [122, 126]. T cell polarisation towards a Th2 or Th9 phenotype is driven by IL-4 [127], with Th9 requiring additional TGFβ signals [126]. In addition, ILC2s play critical roles in Th2 cell polarisation in both IL-4-dependent and independent manners thus shaping the overall type 2 inflammatory environment [122]. In the context of cancer, the type 2 cytokines IL-4, IL-5, IL-9, and IL-13 along with ILC2s, Th2, and Th9 cells may either promote or inhibit tumorigenesis in a context-dependent manner that involves complex interactions with cancer cells and the constituents of the tumour microenvironment.

### IL-4 and IL-13

**Figure 3:**
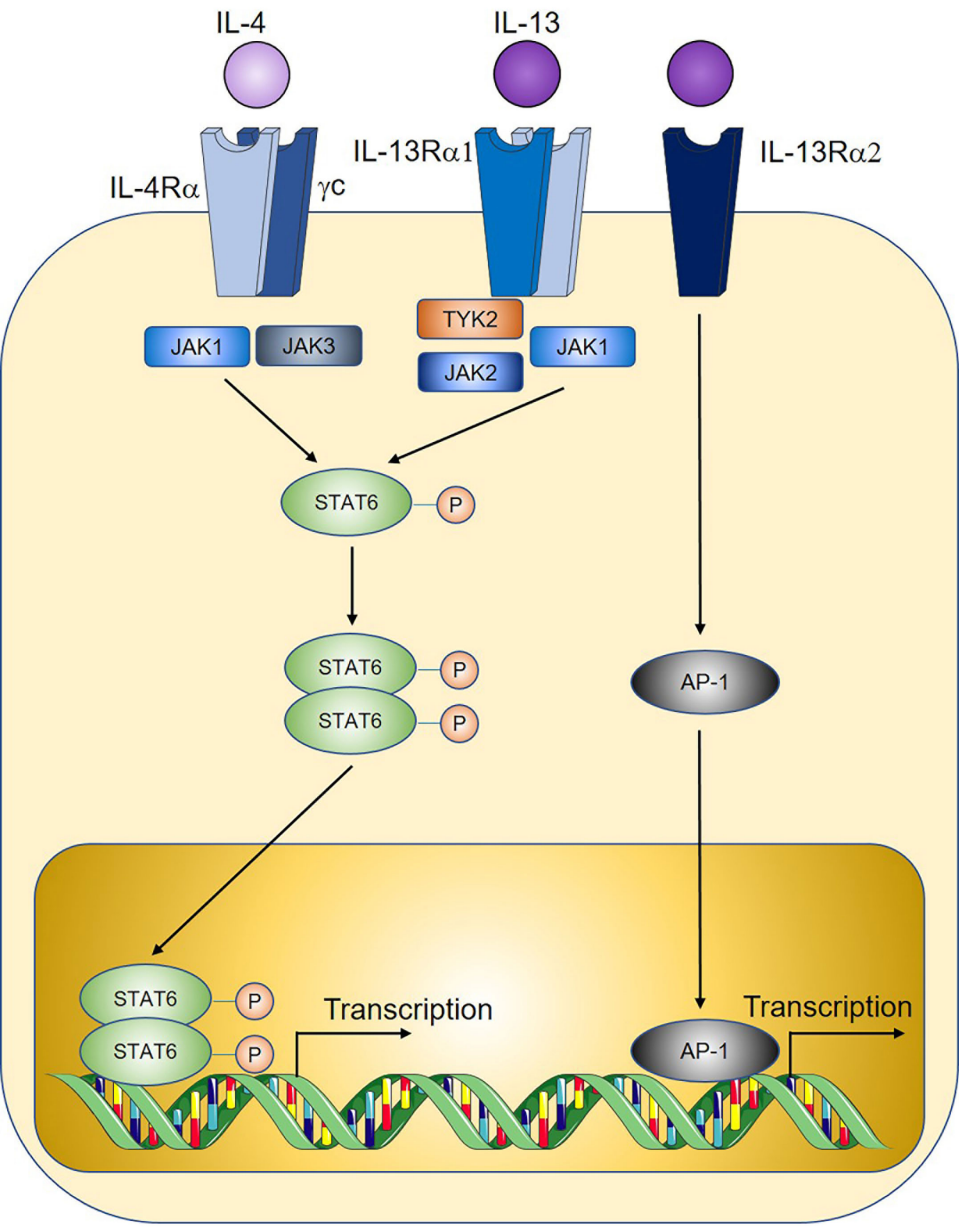
Overview of IL-4 and IL-13 signalling. TYK2: tyrosine kinase 2; AP-1: activator protein 1. Sharp arrows indicate activation; circled P symbols indicate phosphorylation. Figure was created in part using cartoon templates by Servier Medical Art (https://smart.servier.com/), licensed under a Creative Commons Attribution 3.0 Unported License

IL-4, the prototypical type 2 cytokine, exerts its function through binding to IL-4R, which is comprised of the IL-4Rα subunit and the common gamma chain (γc, Figure 3) [128]. Early studies of IL-4 soon after its discovery found that overexpression of IL-4 by tumour cell lines through genetic approaches led to tumour rejection after *in vivo* implantation, consistent with an anti-tumoural role [129–131]. This was observed in a wide variety of tumour cell line cancer models such as renal cell tumour [129], CRC [131], myeloma, and breast cancer [130]. However, the anti-tumoural functions of IL-4 may only occur at supraphysiological levels, as most subsequent studies found that endogenously expressed IL-4 instead exert a predominantly pro-tumoural role. In a mouse model of colitis-associated cancer, genetic IL-4-deficiency (thus removing endogenous IL-4 signals) reduced tumour burden [132], and genetic deletion of STAT6, the downstream signaling mediator for IL-4, similarly reduced tumorigenesis in a model of adenomatous polyposis coli (APC)-mutation-mediated CRC [133]. IL-4 may promote tumorigenesis through multiple proposed mechanisms, including through the induction of Th2 cells which produce other pro-tumoural cytokines such as IL-13 [24]. IL-4-signalling induces alternative activation of macrophages to an M2 tumour-associated macrophage (TAM) phenotype with well-documented pro-tumoural functions in the tumour microenvironment [59, 134]. Tumour cells express elevated levels of IL-4R, and IL-4 signaling reduces cancer cell apoptosis through the upregulation of anti-apoptotic genes [135] while also directly promoting tumour cell proliferation [136]. Together with IL-13, IL-4 may also promote MDSCs to inhibit anti-tumour immunity as shown recently where MDSC-mediated CD8^+^ T cell suppression is critically dependent on IL-4 and IL-13 signalling [24].

Like IL-4, IL-13-signalling is mediated by STAT6, through binding to IL-13R which is comprised of an IL-4Rα subunit shared with the IL-4R, coupled with an IL-13Rα1 subunit that provides specificity (Figure 3) [128]. IL-13 similarly promotes M2 TAM polarisation and MDSC-mediated T cell suppression thereby contributing to tumorigenesis [29, 59, 134]. In a model of sporadic CRC, genetic IL-13 deficiency significantly reduced tumour burden, and adoptive transfer of IL-13^+^ ILC2s compared to IL-13^–^ ILC2s led to enhanced MDSC activation and downstream suppression of anti-tumoural IFNγ^+^ CD8^+^ T cells and Th1 cells [24]. Importantly, when culturing MDSCs with ILC2-derived factors which enhance MDSC-mediated T cell suppression, neutralisation of ILC2-derived IL-4 and IL-13 reprogrammed MDSCs to an anti-tumoural phenotype characterised by enhanced CD8^+^ T cell activation and IFNγ^+^ and granzyme expression. Similarly in a study on bladder cancer, detection of IL-13 in patient urine samples correlated with increased ILC2 and MDSCs prevalence and reduced T cells [137]. IL-13 may also directly promote cancer cell proliferation and metastasis through an alternative IL-13Rα2 (Figure 3) for example in pancreatic cancer [138, 139]. In humans, IL-13R expression is increased in a broad range of solid tumours including CRC, glioblastoma, breast cancer, and pancreatic cancer, and is associated with poor prognosis [140].

### IL-5 and IL-9

IL-5 contributes to type 2 immunity mainly through eosinophils. Eosinophil development is IL-5-dependent, and genetic ablation of the IL-5R subunit IL-5Rα in mice results in defective eosinophils [141]. Likewise, human eosinophil progenitors also express IL-5Rα [142]. Furthermore, IL-5 directly recruits and activates mature eosinophils, and both IL-5 and eosinophils have been implicated in cancer [143, 144]. The majority of animal and human studies seem to suggest an anti-tumoural role of IL-5 and eosinophils, suggesting them as the dominant effectors of anti-tumoural responses by type 2 immunity. Eosinophils can directly induce cancer cell lysis through the release of cytotoxic granules in a human CRC cell line model [145], or indirectly through promoting CD8^+^ T cell-mediated tumour rejection in mouse melanoma [146]. In human patients, increased eosinophils have been associated with improved prognosis across several cancer types including CRC, melanoma, and breast cancer [147–150].

Nevertheless, IL-5 may have pro-tumorigenic roles similar to other type 2 cytokines such as IL-4 and IL-13. A recent study found that IL-5-activated eosinophils upregulated glycolysis, which resulted in depletion of glucose in the tumour microenvironment [151]. This resulted in increased cancer metastasis to the lungs, due to impaired NK cell effector function which is critically dependent on glucose. Similarly, others have found IL-5 and eosinophils to promote lung metastasis by CRC MC38 cells, however instead through the recruitment of Tregs in response to the eosinophil-derived chemokine C-C motif chemokine 22 (CCL22) [144]. In this model, genetic deficiency or antibody-mediated neutralisation of IL-5 reduced metastasis, while adoptive transfer of eosinophils enhanced tumour spread, consistent with a pro-tumoural role. Therefore, the role of IL-5 and eosinophils in tumour development is likely context-dependent and may vary depending on the specific cancer subtype. Indeed, other prognostic studies in humans have found eosinophils to be associated with poor survival in patients with Hodgekin’s lymphoma, leukaemia, and cervical cancer [152–154].

IL-9 is produced by ILC2s and Th9 cells, and like IL-5, has been shown to either promote or inhibit tumorigenesis. In nude mice, exogenous IL-9 treatment inhibited gastric cancer growth [155]. Interestingly, in this model, IL-9 treatment is associated with a reduction in serum IL-4, indicating potential opposing functions. In a mouse model of colitis-associated cancer, IL-9 suppressed tumour growth through CD8^+^ T cell activation [156]. Similarly, findings were observed in melanoma where IL-9 supports anti-tumoural CD4^+^CD8^+^ double-positive T cells directly inducing their proliferation and preventing apoptosis via signaling through the IL-9R [157]. Overexpression of IL-9 by the CT26 CRC cell line genetically led to enhanced CD4^+^ and CD8^+^ T cell recruitment resulting in heightened serum INFγ and tumour lysis [158]. Therefore, although considered as part of type 2 immunity, studies are consistent with IL-9 inducing anti-tumour immunity through cross-activating type 1 immune INFγ producing cells such as CD8^+^ T cells and Th1 cells [155, 158]. Conversely, some studies have found that IL-9 may instead promote tumorigenesis as seen in a heterotypic CT26 implant model of CRC where genetic IL-9 deficiency reduced tumour growth [159]. A potential explanation may be that IL-9 is also a prototypical mast cell growth factor and may exert tumour-promoting properties through mast cells [160]. Mast cells are associated with poor prognosis in a broad range of human cancer types, including melanoma, pancreatic, and prostate cancer [161–163], and can promote cancer angiogenesis through tryptase-mediated extracellular matrix remodelling [164]. Therefore, the role of IL-9 in tumorigenesis is likely dependent on the specific immune cell landscape of the tumour microenvironment depending on the cancer type.

### IL-25 and IL-33: novel players of type 2 immunity in tumorigenesis

IL-25 and IL-33 are well-established type 2 immune-related cytokines with recently discovered roles in cancer immunity. Both are potent inducers of type 2 immunity with some degree of overlap in function, despite belonging to distinct cytokine families [165]. Specifically, IL-33 and IL-25 directly activate ILC2s and stimulate the release of type 2 cytokines, and the differential sensitivities of tissue-specific ILC2s to these cytokines underlie their functional differences and relative importance in inducing type 2 immunity at different organs [166]. For example, ILC2s in the intestines express high levels of IL-25R, while expression of IL-33R component suppression of tumorigenicity 2 (ST2) is minimal [167]. IL-33 also has additional roles in stimulating Tregs and Th17 cells, the latter likely in a mast cell-dependent manner [168–171]. These may underlie the differential roles of IL-33 and IL-25 in different cancer types depending on the tissue site.

The roles of IL-33 and IL-25 in CRC have recently been reviewed [31]. Briefly, IL-25 is produced by rare tuft cells in the intestines and may promote CRC tumorigenesis through directly inducing tumour stemness or indirectly via stimulation of ILC2s which activates MDSCs [24, 172, 173]. Critically, therapeutic blockade of the IL-25-ILC2-MDSC axis increased IFNγ^+^ expressing CD8^+^ T cells and Th1 cells and significantly reduced CRC burden. In human CRC patients, increased tumour *IL-25* expression is associated with poor prognosis, indicating that blocking IL-25 signalling may be a potential novel therapeutic option. Conversely, others have found that IL-25 treatment can reduce subcutaneous tumour growth across a broad range of implanted cancer cell lines in immunodeficient mice [174], most likely through directly inducing apoptosis as shown in breast cancer cells [175]. Therefore, any therapeutic attempts utilising IL-25 to treat cancer will need to be directed to tumour cells and must be used with caution given the potential to elicit pro-tumoural immune responses in the tumour microenvironment.

IL-33 may similarly promote or inhibit cancer in a context-dependent manner. Genetic deficiency of ST2 enhanced NK cell effector function resulting in reduced 4T1 breast cancer growth and metastasis in mice [176]. Others have found IL-33 to stimulate ILC2-mediated NK cell suppression in lung cancer [151]. Similarly, IL-33 administration increased tumour-infiltrating IL-13^+^ ILC2s, MDSCs, and Tregs, and correlated with reduced NK cell cytotoxicity and accelerated tumour growth and metastasis to the lung and liver [177]. Mechanistically, IL-33 can promote metastasis via the induction of desmoplastic reactions by activating cancer-associated fibroblasts [178]. Furthermore, both IL-33 and IL-25 have been shown to promote angiogenesis which also contributes to metastasis, and genetic deletion of ST2 reduced VEGF expression and tumour burden in mouse breast cancer [179–181]. IL-33 can also promote tumorigenesis through stimulating mast cells and Tregs, as seen in mouse models of CRC [182, 183].

Currently, there are no clinical trials related to IL-33 or IL-25 for cancer treatment. Nevertheless, given their overarching effects on tumour immunity, acting upstream of the main effector type 2 cytokines IL-4, IL-5, IL-9, and IL-13 and T cells, the prospect of therapeutically targeting these cytokines is promising. For example, MDSCs have been shown to exert therapeutic resistance across all cancer treatment modalities including surgery, chemotherapy, radiotherapy, and immunotherapy across a broad range of cancer types [184–188]. Combination therapies through concomitant IL-25-signalling blockade may effectively deplete MDSCs by inhibiting the IL-25-ILC2-MDSC axis thereby potentiating existing cancer therapies [24].

## Type 2 cytokines in immunotherapy

Unlike type 1 immune cytokines, there are few clinical trials exploring the therapeutic efficacy of targeting type 2 cytokines. This is likely due to the less consistent roles of type 2 immunity in cancer where they may either promote or suppress tumorigenesis depending on specific contexts. In the case of IL-4, preclinical studies as discussed above indicate that supraphysiologic levels of exogenous IL-4 may induce tumour rejection, while endogenous IL-4 typically promotes tumour progression [129–133]. Therefore, potential strategies for cancer treatment may involve injecting high levels of IL-4 or blocking existing IL-4-signalling in patients. Human cancer studies have found IL-4R expression to be elevated in many tumours types such as bladder, lung, breast, liver, and prostate cancer [135, 189, 190], suggesting that IL-4-signalling can be selectively targeted therapeutically for cancer treatment. A phase I clinical trial of 17 patients with solid tumours found that IL-4 treatment is limited by toxicities, with symptoms including weight gain, effusions, rash, peripheral oedema, and oliguria [191]. Importantly, whether the maximally tolerated dose of IL-4 injected in patients is sufficient to elicit anti-tumour properties is unclear. In a phase II trial of advanced renal cell carcinoma where 49 patients were treated with subcutaneous injection of IL-4 at 5 mcg/kg per day for 28 days, no complete or partial responses were observed [192]. In a similar phase II trial by the same group but instead in melanoma patients, only 1 patient responded to IL-4 therapy out of the 34 treated patients [193]. Another phase II study also concluded no benefit of IL-4 treatment at the maximum tolerated dose for melanoma or renal cell carcinoma [194]. Currently, there are no ongoing oncology clinical trials involving IL-4. Future clinical trials involving IL-4 administration will have to overcome the hurdle of therapeutic toxicities in order to achieve a clinically effective dose, or alternatively through monoclonal antibodies neutralising endogenous pro-tumoural IL-4 or blocking the IL-4R.

Similar to IL-4, currently there are no ongoing clinical trials involving IL-5 or IL-9 in cancer treatment, likely a reflection of our current inadequate understanding of whether they would promote or inhibit cancer under different contexts. Furthermore, there are few studies that directly assessed the expression of IL-5, IL-9, or their receptors in human cancer. IL-9-producing Tregs have been detected in human non-small cell lung cancer samples, while others have found that IL-9R expression is significantly enhanced in endometrial cancer compared to other cancer types such as renal or breast cancers [195, 196]. Conversely, increased breast cancer IL-5 expression is associated with metastasis and poor prognosis [197], while others have found that tumour Th2 cell-derived IL-5 may instead enhance response to immune checkpoint inhibitors in breast cancer [198]. Future trials targeting these cytokines should therefore focus on cancer types where these cytokines and respective receptors are readily detected. On the other hand, studies consistently showed IL-13 to exert pro-tumoural properties, and in humans, particularly, through the alternative IL-13Rα2 [138, 140]. Inhibiting IL-13 has many theoretical benefits, including reducing MDSCs and M2 TAMs, while promoting anti-tumour IFNγ^+^ and T cell infiltration [24, 59, 134]. This can be achieved through monoclonal antibody-mediated neutralisation of IL-13 or blocking the IL-13R, or alternatively through recently developed IL-13Rα2-targeting chimeric antigen receptor (CAR)-T cells [199]. CARs are genetically engineered receptors that redirect T cells to recognise and eliminate cells expressing the target antigen, in this case, IL-13Rα2. In gliomas, IL-13Rα2 expression is only detected in tumours and not normal brain tissue [200]. Currently, there are six ongoing clinical trials utilising IL-13Rα2-targeting CAR-T cells, either alone or in combination with chemotherapy or checkpoint inhibitor immunotherapy (Table 3). Chemotherapy can induce antigen release while checkpoint inhibitors revitalise exhausted T cells [201], theoretically potentiating CAR-T cell therapy. Furthermore, in one study, the IL-2 cytokine is also added which has a major role in inducing T cell proliferation [202] and may further enhance IL-13Rα2-targeting CAR-T cell efficacy. In addition to directly killing tumour cells, IL-13Rα2-targeting CAR-T cells may eliminate other IL-13-responsive cells in the tumour microenvironment contributing to therapeutic efficacy.

**Table 3:** A comprehensive list of ongoing oncology clinical trials involving IL-13

**Cytokine**	**Clinical trial ID**	**Phase**	**Number of patients**	**Cancer type**	**Treatment**	**Status**	**Estimated study completion date**
IL-13	NCT02208362	I	82	Recurrent or refractory glioma	IL-13Rα2-specific Hinge-optimized 4-1BB-co-stimulatory CAR/truncated CD19-expressing autologous T cells	Active	June 2023
IL-13	NCT04119024	I	24	Melanoma	IL-13Rα2-specific Hinge-optimized 4-1BB-co-stimulatory CAR/truncated CD19-expressing autologous naive and memory T cells plus cyclophosphamide, fludarabine, and IL-2	Recruiting	October 2025
IL-13	NCT04661384	I	30	Ependymoma, glioblastoma, medulloblastoma, leptomeninges cancer	IL-13Rα2-specific Hinge-optimized 4-1BB-co-stimulatory CAR truncated CD19-expressing autologous T-lymphocytes	Recruiting	November 2025
IL-13	NCT04510051	I	18	Brain cancer	IL-13Rα2-specific Hinge-optimized 4-1BB-co-stimulatory CAR truncated CD19-expressing autologous T-lymphocytes plus cyclophosphamide and fludarabine	Recruiting	September 2023
IL-13	NCT04003649	I	60	Recurrent or refractory glioblastoma	IL-13Rα2-specific Hinge-optimized 4-1BB-co-stimulatory CAR/truncated CD19-expressing autologous naive and memory T cells plus ipilimumab and nivolumab	Recruiting	December 2024
IL-13	NCT05168423	I	18	Glioblastoma	CAR-T-EGFR-IL-13Rα2 plus cyclophosphamide and fludarabine	Not yet recruiting	December 2039

EGFR: epidermal growth factor receptor

## Conclusions

Cytokines play overarching roles in shaping tumour immunity with broad influence on the tumour microenvironment and are attractive targets for cancer immunotherapy. Understanding how each cytokine reacts and influences the tumour microenvironment of different cancer types is critical in order to achieve beneficial therapeutic outcomes, especially as the same cytokine may have entirely opposing functions in different cancers. Overall, exogenous administration of type 1 immune cytokines for cancer treatments, particularly IFNγ and IL-12, are promising but may be limited by toxicities. Ongoing clinical trials will continue to investigate different modalities of administration to minimise iatrogenic harm while maximising treatment efficacy. A better contextual understanding of type 2 immune cytokines, in particular IL-5 and IL-9, will open new avenues for treatment testing in clinical trials. The roles of cytokines in cancer pathways are continuingly being discovered and will have great impacts on future cancer therapy.

## Abbreviations

CAR: chimeric antigen receptor
CRC: colorectal cancer
DCs: dendritic cells
IFNGR: interferon γ receptor
IFNγ: interferon γ
IL-12: interleukin-12
IL-12R: interleukin-12 receptor
ILC2s: group 2 innate lymphoid cells
ISG: interferon-stimulated gene
JAK1: Janus kinase 1
MDSCs: myeloid-derived suppressor cells
NK: natural killer
pAPCs: professional antigen-presenting cells
PD-L1: programmed death-ligand 1
ST2: suppression of tumorigenicity 2
STAT1: signal transducer and activator of transcription 1
TAM: tumour-associated macrophage
TGFβ: transforming growth factor β
Th1: T helper 1
TNFR2: tumour necrosis factor receptor type 2
TNFα: tumour necrosis factor α
Treg: regulatory T

## Declarations

### Copyright

© The Author(s) 2023.
